# Supplementary material for: Association between atrial fibrillation and risk of end-stage renal disease among adults with diabetes mellitus
Source: PLoS One. 2022 Aug 26;17(8):e0273646. doi: 10.1371/journal.pone.0273646 (PMC9417190; doi:10.1371/journal.pone.0273646)
Supplement: Attachment — Submitted filename: Response to reviewers.docx [file pone.0273646.s003.docx]

**Response to Reviewers’ comments**

**Ref.: PONE-D-21-17026**

Reviewer #1: Thank you for letting me review given article. I have no further comments. Comments were addressed as fully as possible.

**Reply:** Thank you.

Reviewer #2: I appreciate that the authors' responses to my comments. However, I still have a few concerns that might be helpful in strengthening the manuscript:

The authors indicated that some risk factors have missing values which result in smaller sample size and a loss of statistical power. Given presence of missing values of important covariates for analysis, it would be interesting to conduct multiple imputations (MI). MI will allow a better statistical power and adjustment for more appropriate set of covariates to draw conclusions.
**Reply:** Thank you for your comment. MI is a tool to deal the missing research data essentially that happens when people fail to respond to a survey. The NHIRD is a registered claims dataset that has fewer missing data problem. In the previous study was showed that a rule of thumb blow of 5% missingness was not necessary to multiple imputation.^1^ So that MI might not be suitable to use in the study that is not somewhat true to the original in order to add the statistic power. On the other hand, we chose the PS matching in the [statistical](https://en.wikipedia.org/wiki/Statistics) analysis of [observational data](https://en.wikipedia.org/wiki/Observational_study) to construct an artificial control group by matching each treated unit with a non-treated unit of similar characteristics that attempts to [reduce](https://en.wikipedia.org/wiki/Estimation_theory) bias arises.

Reference:

1. Jakobsen JC, Gluud C, Wetterslev J, Winkel P. When and how should multiple imputation be used for handling missing data in randomised clinical trials - a practical guide with flowcharts. BMC Med Res Methodol. 2017;17(1):162. Epub 2017/12/07. doi: 10.1186/s12874-017-0442-1. PubMed PMID: 29207961; PubMed Central PMCID: PMCPMC5717805.

I see that the authors performed competing risk analysis. I would suggest including the description of methods used to perform the competing risk analysis and provide these results in the manuscript.
**Reply**: Thank you for your comment. Please see Main document_Clean file. We have add the description of competing risk analysis in the Methods. (Please see P6L15-17)

While the authors indicated that groups were matched using PS, Table 1 shows that there are differences in some of the important covariates. I do understand that the authors included these covariates later in the multivariable regression. However, why would the authors perform PS matching (while missing some of important covariates) and then including them in the final analysis. Would it be easier to just conduct multivariable analysis without matching? And then conduct analysis on the matched groups? I am just struggling to understand with concepts why the authors done the analysis this way.

**Reply:** Thank you for your kindly comment. In the [statistical](https://en.wikipedia.org/wiki/Statistics) analysis of [observational data](https://en.wikipedia.org/wiki/Observational_study), PS matching is a quasi-experimental method such as the investigator uses statistical techniques to construct an artificial control group by matching each treated unit with a non-treated unit of similar characteristics that attempts to [reduce](https://en.wikipedia.org/wiki/Estimation_theory) bias arises. Because a difference in the treatment outcome between treated and untreated groups may be caused by a factor that predicts treatment rather than the treatment itself. We had used this statistical analysis published some journal articles based on NHIRD^1-8^. In our earliest study^8^, we used PS matching among patients with peritoneal dialysis and hemodialysis, because of patients treated with PD were different from patients treated with HD in terms of health status. The PD and HD cohorts were homogenous in sociodemographic characteristics and comorbid conditions at the baseline after using the propensity score-matched design, which reduces the selection bias of samples and potential confounding effect. The general rule of practice in the previous study was showed that the covariates can be added into a regression adjustment that could be dramatically remove residual confounding bias if remaining imbalance after PS matching.^9^

References:

1. Chen YY, Fan HC, Tung MC, Chang YK. The association between Parkinson's disease and temporomandibular disorder. PLoS One. 2019; 14(6):e0217763.
2. Lu CW, Chang YK, Lee YH, Kuo CS, Chang HH, Huang CT, Hsu CC, Huang KC. Increased Risk for Major Depressive Disorder in Severely Obese Patients after Bariatric Surgery - a 12-year Nationwide Cohort Study. Ann Med. 2018; 13:1-30. doi: 10.1080/07853890.2018.1511917.
3. Chang HH, Chang YK, Lu CW, Huang CT, Chien CT, Hung KY, Huang KC, Hsu CC. Statins Improve Long Term Patency of Arteriovenous Fistula for Hemodialysis. Sci Rep. 2016; 6:22197. doi: 10.1038/srep22197.
4. Lu CW, Chang YK, Chang HH, Kuo CS, Huang CT, Hsu CC, Huang KC. [Fracture Risk After Bariatric Surgery: A 12-Year Nationwide Cohort Study.](http://www.ncbi.nlm.nih.gov/pubmed/26632892) Medicine (Baltimore). 2015; 94(48): e2087.
5. Hung SC, Chang YK, Liu JS, Hsu CC, Tarng DC. [Mortality and metformin use in patients with advanced chronic kidney disease.](http://www.ncbi.nlm.nih.gov/pubmed/26298571) Lancet Diabetes Endocrinol. 2015 Aug;3(8):605-14. doi: 10.1016/S2213-8587(15)00123-0.
6. Hsu CC, Wang H, Hsu YH, Chuang SY, Huang YW, Chang YK, Liu JS, Hsiung CA, Tsai HJ. [Use of Nonsteroidal Anti-Inflammatory Drugs and Risk of Chronic Kidney Disease in Subjects With Hypertension: Nationwide Longitudinal Cohort Study.](http://www.ncbi.nlm.nih.gov/pubmed/26169048) Hypertension. 2015; 66(3): 524-33. doi: 10.1161/HYPERTENSIONAHA. 114.05105.
7. Kuo KL, Hung SC, Liu JS, Chang YK, Hsu CC, Tarng C. Iron supplementation associates with low mortality in pre-dialyzed advanced chronic kidney disease patients receiving erythropoiesis-stimulating agents: a nationwide database analysis. Nephrol Dial Transplant. 2015; 30(9): 1518-25. doi: 10.1093/ndt/gfv085.
8. Chang YK, Hsu CC, Hwang SJ, Chen PC, Huang CC, Li TC, Sung FC. A Comparative Assessment on Survival between Propensity Score Matched Patients with Peritoneal Dialysis and Hemodialysis in Taiwan. Medicine. 2012; 91(3): 144-151. doi: 10.1097/MD.0b013e318256538e.
9. Nguyen TL, Collins GS, Spence J, Daures JP, Devereaux PJ, Landais P, et al. Double-adjustment in propensity score matching analysis: choosing a threshold for considering residual imbalance. BMC Med Res Methodol. 2017;17(1):78. Epub 2017/04/30. doi: 10.1186/s12874-017-0338-0. PubMed PMID: 28454568; PubMed Central PMCID: PMCPMC5408373.
